# Corticotropin releasing factor excites neurons of posterior hypothalamic nucleus to produce tachycardia in rats

**DOI:** 10.1038/srep20206

**Published:** 2016-02-01

**Authors:** He-Ren Gao, Qian-Xing Zhuang, Bin Li, Hong-Zhao Li, Zhang-Peng Chen, Jian-Jun Wang, Jing-Ning Zhu

**Affiliations:** 1 State Key Laboratory of Pharmaceutical Biotechnology and Department of Biological Science and Technology, School of Life Sciences, Nanjing University, 163 Xianlin Avenue, Nanjing 210023, China

## Abstract

Corticotropin releasing factor (CRF), a peptide hormone involved in the stress response, holds a key position in cardiovascular regulation. Here, we report that the central effect of CRF on cardiovascular activities is mediated by the posterior hypothalamic nucleus (PH), an important structure responsible for stress-induced cardiovascular changes. Our present results demonstrate that CRF directly excites PH neurons via two CRF receptors, CRFR1 and CRFR2, and consequently increases heart rate (HR) rather than the mean arterial pressure (MAP) and renal sympathetic nerve activity (RSNA). Bilateral vagotomy does not influence the tachycardia response to microinjection of CRF into the PH, while β adrenergic receptor antagonist propranolol almost totally abolishes the tachycardia. Furthermore, microinjecting CRF into the PH primarily increases neuronal activity of the rostral ventrolateral medulla (RVLM) and rostral ventromedial medulla (RVMM), but does not influence that of the dorsal motor nucleus of the vagus nerve (DMNV). These findings suggest that the PH is a critical target for central CRF system in regulation of cardiac activity and the PH-RVLM/RVMM-cardiac sympathetic nerve pathways, rather than PH-DMNV-vagus pathway, may contribute to the CRF-induced tachycardia.

Corticotropin releasing factor (CRF), also known as corticotropin-releasing hormone, is a critical neuropeptide responsible for initiating coordinated autonomic, endocrine and behavioral responses to stress^1^,^2^. Both acute and chronic stresses cause changes in heart rate (HR) and arterial pressure (AP)^3^,^4^,^5^, indicating a close relationship between the CRF system and cardiovascular regulation. In fact, intracerebroventricular injection of CRF related peptides increases HR, cardiac output, and mean arterial pressure (MAP)^6^,^7^,^8^, whereas systemic administration of CRF or urocortin 1, an endogenous member of CRF family, remarkably decreases MAP and increases superior mesenteric artery flow^9^. Urocortin has also been shown to induce positive inotropic effect in heart^10^,^11^. Thus, CRF related peptides may profoundly modulate cardiovascular activities via both central and peripheral mechanisms.

Two major subtypes of G-protein-coupled receptors for CRF related peptides have been identified, CRFR1 and CRFR2^2^,^12^. Both of them are expressed in the central nervous system^2^,^13^,^14^ and periphery^2^,^15^ and mediate cardiovascular effects of the CRF system. In CRFR1 knockout mice, an abnormally high cardiac noradrenergic activity following stress induced by morphine withdrawal, which is observed on wildtype mice, is inhibited^16^. Moreover, systemic administration of urocortin 1 fails to decrease MAP in CRFR2 knockout mice, whereas wildtype mice show a marked reduction^17^,^18^. Although the peripheral effect of CRF on cardiovascular activities primarily mediated by CRFR2 has been well studied, the central pathway and mechanism underlying the modulation of CRF system on cardiovascular activities is still largely unknown.

The posterior hypothalamic nucleus (PH) is an important center for cardiovascular regulation. Electrical^19^ or chemical stimulation^20^,^21^ of the PH increases AP, HR and sympathetic nerve activity. Recently, it has been reported that in patients, chronic deep brain stimulation of the PH is associated with an enhanced sympathoexcitatory drive on the cardiovascular system^22^. Intriguingly, the PH holds a key position in the generation of stress-induced cardiovascular changes^23^,^24^. Therefore, in the present study, we examined the contribution of the PH in the central effects of CRF on cardiovascular activities and the underlying mechanisms using electrophysiological techniques combined with molecular and immunostaining methods.

## Results

### Expression of CRFR1 and CRFR2 in the PH

**Figure 1:**
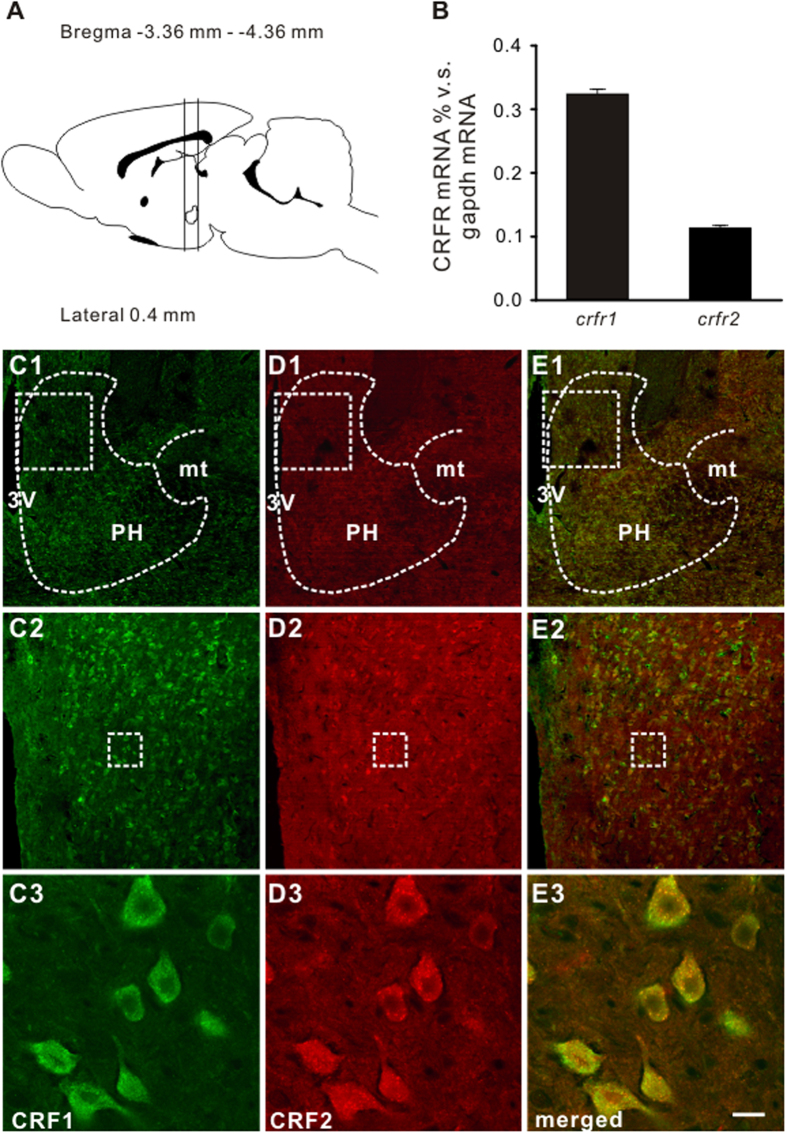
Quantitative real-time RT-PCR and immunofluorescence results showing that CRFR1 and CRFR2 were expressed in the rat PH. (**A**) Sagittal view of the rat brain (Paxinos and Watson, 2007), localizing the PH between −3.36 and −4.36 mm from the bregma. (**B**) Bar graphs showing relative expression of CRFR1 and CRFR2 in the rat PH. (**C1–C3**) CRFR1 was present in the rat PH. (**D1,D2**) CRFR2 was present in the PH. (**E1,E2**) CRFR1 and CRFR2 were co-localized in the same PH neurons. Scale bars: (**C1**), (**D1**) and (**E1**), 550 μm; (**C2**), (**D2**) and (**E2**), 160 μm; (**C3**), (**D3**) and (**E3**), 20 μm. 3V, 3th ventricle; mt, mammillothalamic tract; PH, posterior hypothalamic nucleus.

We assessed the expressions of CRFR1 and CRFR2 mRNAs in the PH in rats (*n* = 5) by quantitative real-time RT-PCR. The rat PH tissues, localized between −3.36 and −4.36 mm from bregma (Fig. 1A), were extracted according to the rat brain atlas^25^. As shown in Fig. 1B, the *crhr1* mRNA % v.s. *gapdh* mRNA was 0.3241 ± 0.0073, whereas the *crhr2* mRNA % v.s. *gapdh* mRNA was 0.1136 ± 0.0038. Thus, both CRFR1 and CRFR2 mRNAs were detected in the rat PH.

To further map the distributions of CRFR1 and CRFR2 in the PH, we performed immunofluorescence of the rat brain slices (*n* = 5) containing the PH with an antibody against CRFR1 and CRFR2, respectively. We found that both CRFR1 and CRFR2 were not only localized in the PH (Fig. 1C1–C3 and D1–D3), but also co-localized in the same PH neurons (Fig. 1E1, E2 and E3).

### CRF remarkably excites PH neurons via CRFR1 and CRFR2

**Figure 2:**
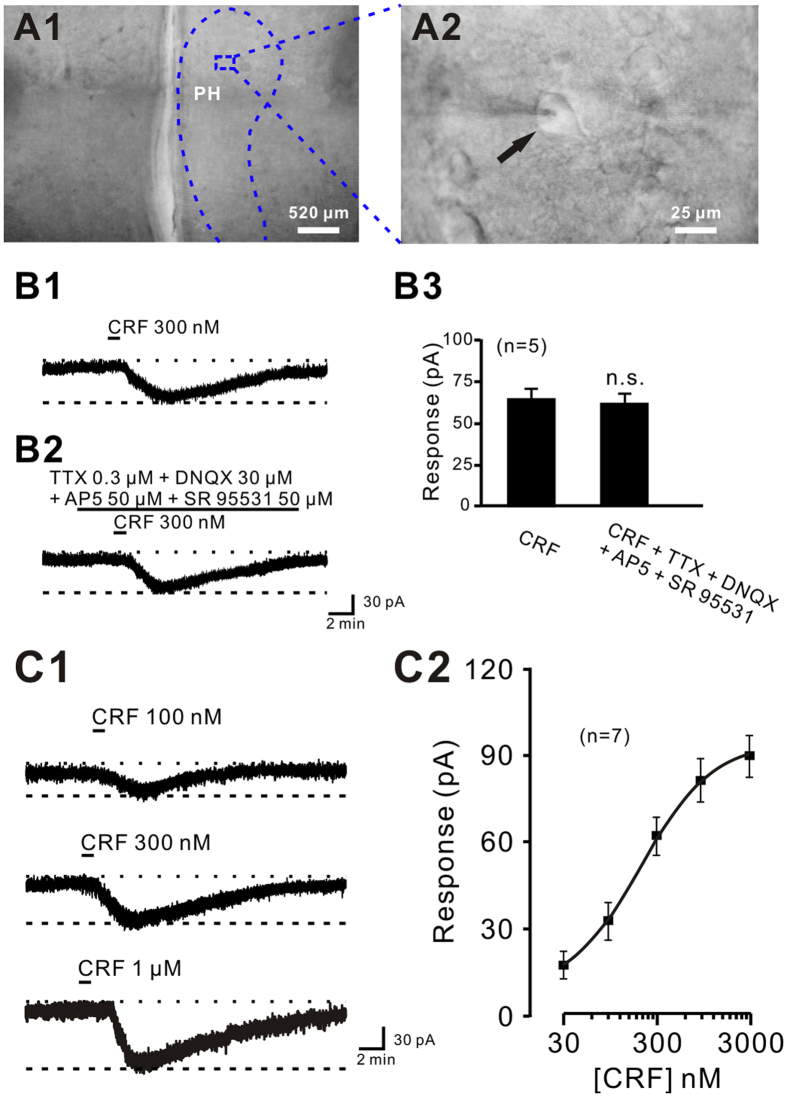
CRF excited PH neurons with a direct postsynaptic manner. (**A1**,**A2**) Graph of brain section shows the area of PH investigated in this study. (**B1**,**B2**) CRF still induce an inward current when perfusing the slice with TTX. (**B3**) Group data of the 5 recorded PH neurons in rats. (**C1**) CRF concentration-dependently excited PH neurons. (**C2**) Concentration-response curves for CRF on the 7 recorded PH neurons in rats shows the mean EC50 of 176.5 nM. In this and the following figures, the short horizontal bars above the data indicate the period of application of CRF or CRF receptor agonist, and the long horizontal bars indicate the exposure of the slice to TTX and CRF receptor antagonist.

We recorded a total of 37 PH neurons in rats (*n* = 5) with the input resistance higher than 300 MΩ and the capacitance of 113.5 ± 16.5 pF in the present study by carrying out whole-cell voltage clamp recordings on brain slices. An example illustrating the location (Fig. 2A1) and morphology (Fig. 2A2) of one recorded neuron in the PH was shown in Fig. 2. We found that CRF (300 nM) significantly elicited an inward current (65.6 ± 5.5 pA) on the recorded PH neurons (*n* = 5; Fig. 2B1). Since there are extensive glutamatergic and GABAergic neurotransmissions in the PH^26^,^27^, 0.3 μM tetrodotoxin (TTX) combined with 30 μM 6,7-dinitroquinoxaline-2,3-dione (DNQX, selective non-NMDA receptor antagonist), 50 μM D-(-)-2-amino-5-phosphonopentanoic acid (AP5, potent, selective NMDA receptor antagonist), and 50 μM 6-imino-3-(4-methoxyphenyl)-1(6H)-pyridazinebutanoic acid hydrobromide (SR 95531, selective GABA_A_ receptor antagonist) did not influence the CRF-induced inward current on the PH neurons (63.8 ± 5.3 pA, *n* = 5, *P* = 0.569; Fig. 2B2 and B3). The result substantially demonstrates that the excitatory effect of CRF on PH neurons is a direct postsynaptic action. In addition, the CRF-induced direct excitation was concentration-dependent (Fig. 2C1). Application of 100 nM, 300 nM, and 1 μM CRF elicited an inward current of 36.5 ± 4.1 pA, 60.8 ± 4.5 pA, and 85.6 ± 5.1 pA, respectively, on the same recorded PH neurons. Fitting the concentration-response curves from 7 PH neurons yielded that the mean concentration of CRF for half-maximal activation (EC50) was 176.5 nM (Fig. 2C2).

**Figure 3:**
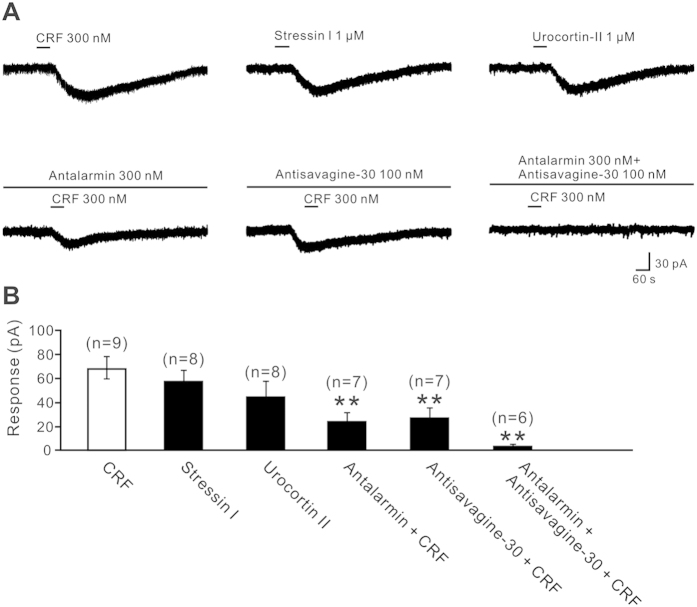
CRF excited PH neurons via CRFR1 and CRFR2. (**A**) CRF excited PH neurons, and highly selective agonists for CRFR1 and CRFR2, stressin I and urocortin II, respectively, mimicked the excitatory effect of CRF on PH neurons. (**B**) Selective CRFR1 antagonist antalarmin and selective CRFR2 antagonist antisavagine-30 partly blocked the excitation of PH neurons induced by CRF, and in combination with antalarmin and antisavagine-30 totally blocked the neuronal excitation induced by CRF. (**C**) Group data of the tested PH neurons in rats. Statistical significance was determined using Student’s t test. **P < 0.01.

Given that CRF exerts its physiological action via two distinct receptor subtypes^15^, CRFR1 and CRFR2, we used selective receptor agonists and antagonists to examine which CRF receptor/receptors mediated the CRF-induced excitation on PH neurons. As shown in Fig. 3, stressin I (1 μM) and urocortin II (1 μM), highly selective agonists for CRFR1 and CRFR2, respectively, mimicked the 300 nM CRF-induced inward current (58.7 ± 5.2 pA and 44.6 ± 4.1 pA, respectively; *n* = 8) on PH neurons. On the other hand, separate application of antalarmin hydrochloride and antisavagine-30, highly selective antagonists for CRFR1 and CRFR2, respectively, partly blocked the CRF-induced inward current from 70.2 ± 6.5 pA to 22.5 ± 2.1 pA and 26.7 ± 3.1 pA, respectively (*n* = 7, *P* < 0.01). Furthermore, combined application of the two antagonists nearly totally blocked the inward current (2.1 ± 0.4 pA; *n* = 6, *P* < 0.01). These results strongly suggest that both CRFR1 and CRFR2 are involved in the CRF-induced postsynaptic excitation on PH neurons.

### Microinjection of CRF into the PH significantly increases HR rather than MAP via the activation of CRFR1 and CRFR2

**Figure 4:**
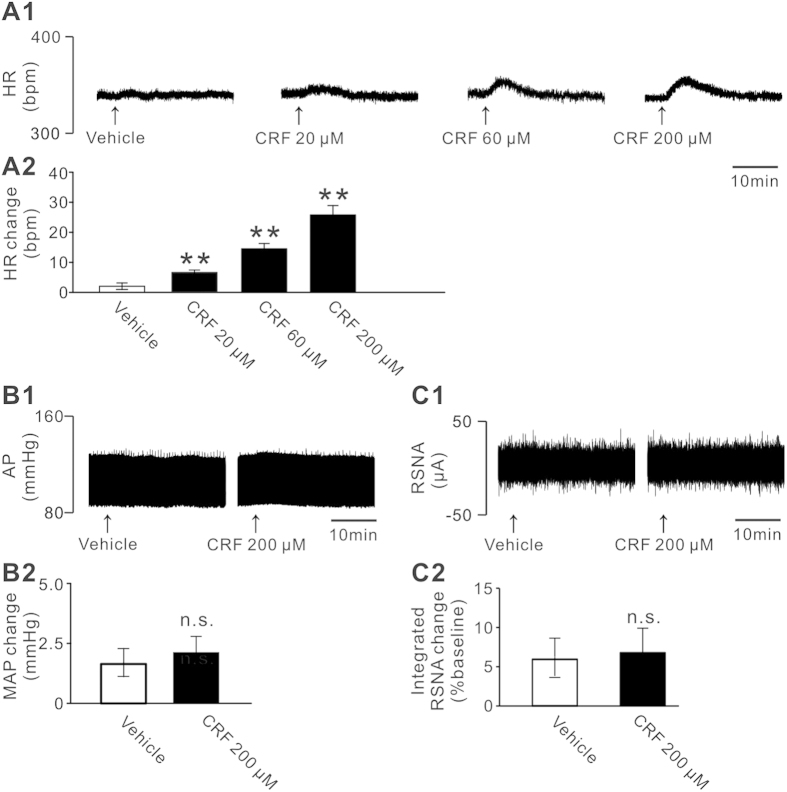
Effect of microinjection of CRF into the PH on the cardiovascular activity. (**A1**) Concentration-dependent increases in HR induced by microinjection of CRF into the PH. (**A2**) Group data showing the changes of average peak HR evoked by CRF or vehicle. (**B1**,**C1**) Changes of AP and RSNA induced by microinjection of CRF into the PH. (**B2**,**C2**) Group data showing the changes of MAP and integrated RSNA evoked by CRF or vehicle in rats. Statistical significance was determined using Student’s t test. n.s. no significance, **P < 0.01.

Next, whether the excitatory action of CRF on PH neurons influences cardiovascular activities *in vivo* was assessed. We found that microinjection of 20, 60 and 200 μM CRF into the rat PH elicited a remarkable increase in average peak HR (6.3 ± 0.8, 13.8 ± 1.7 and 25.8 ± 3.1 bpm, respectively) in a concentration-dependent manner, compared to no change (3.2 ± 0.7 bpm) following injection of normal saline (*n* = 8, *P* < 0.01; Fig. 4A1,4A2). However, microinjecting CRF (up to 200 μM) into the PH did not influence MAP (Fig. 4B1 and B2) and RSNA (Fig. 4C1 and C2). The changes in MAP after injection of CRF or vehicle were 2.8 ± 0.8 mmHg and 1.7 ± 0.8 mmHg (*n* = 8, *P* = 0.36), respectively. And the change ratios of integrated RSNA were 6.3 ± 2.9% *v.s.* 6.1 ± 2.4% (*n* = 8, *P* = 0.16). The data indicate that the PH contributes to potentiation effect of CRF on HR rather than AP and RSNA.

**Figure 5:**
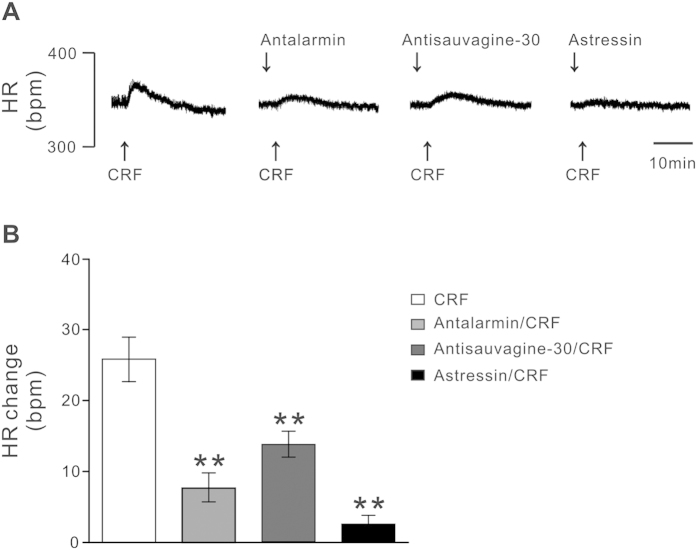
The effect of microinjection of antalarmin, antisauvagine-30, and astressin into the PH on the increased HR response by microinjection of CRF into the PH. (**A**) Changes in HR, evoked by microinjection of CRF into the PH, and the CRF-induced changes after microinjection of antalarmin, antisauvagine-30, and astressin. (**B**) Group data showing the CRF-induced changes in average peak HR in rats before and after microinjection of CRF receptor antagonists. Statistical significance was determined using Student’s t test. *P < 0.05, **P < 0.01.

Given an involvement of both CRF receptors in the excitation of CRF on PH neurons, we further addressed whether CRFR1 and CRFR2 in the PH co-mediate the enhancement effect of CRF on HR. In this experiment, selective CRFR1 antagonist antalarmin hydrochloride or selective CRFR2 antagonist antisauvagine-30 was microinjected into the PH, followed within 2 min by a microinjection of CRF at the same site. As shown in Fig. 5, blockade of either CRFR1 or CRFR2 in the PH partly reduced the tachycardic effect induced by CRF. The increase in average peak HR induced by CRF decreased from 25.8 ± 3.1 bpm to 7.8 ± 2.1 bpm (*n* = 5, *P* < 0.05) and 13.8 ± 1.9 bpm (*n* = 5, *P* < 0.05), respectively. Moreover, microinjection of broad-spectrum CRF receptor antagonist astressin almost totally blocked the tachycardic response to microinjection of CRF into the PH (the increase in average peak HR decreased to 2.6 ± 1.3 bpm; *n* = 5, *P* < 0.01), suggesting that both CRFR1 and CRFR2 in the PH are involved in the tachycardic effect of CRF.

### Effect of vagotomy and blockade of CSNA on the CRF-induced tachycardia

**Figure 6:**
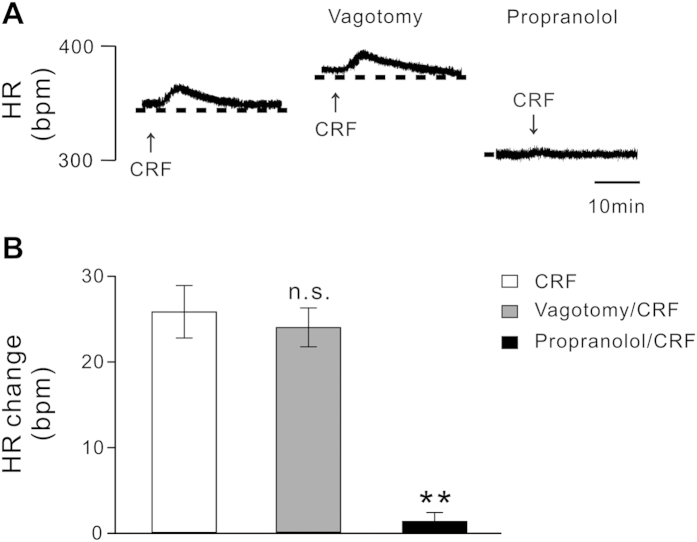
Effect of vagotomy and blockade of cardiac sympathetic nerve activity (CSNA) on the increased HR response by microinjection of CRF into the PH. (**A**) Changes in HR, evoked by microinjection of CRF into the PH, before and after intravenous administration of vagotomy and propranolol (blockade of CSNA). (**B**) Group data showing the CRF-induced changes in average peak HR in rats before and after vagotomy and blockade of CSNA. Statistical significance was determined using Student’s t test. n.s. no significance, **P < 0.01.

To clarify the peripheral mechanism underlying the tachycardia effect of CRF mediated by the PH, we determined whether the vagus nerve or cardiac sympathetic nerve was involved in. As shown in Fig. 6, bilateral vagotomy increased the baseline of HR; however, it only slightly reduced the tachycardic response elicited by microinjection of CRF into the PH. The increases in HR induced by microinjection of CRF before and after the vagotomy were 25.8 ± 3.1 bpm and 22.0 ± 2.1 bpm, respectively (*n* = 5, *P* = 0.56). On the other hand, blocking CSNA by intravenous injection of propranolol almost abolished the tachycardic response elicited by microinjection of CRF into the PH. The increase in HR induced by microinjection of CRF after the blockade of CSNA reduced to 1.4 ± 1.0 bpm (*n* = 5, *P* < 0.01; Fig. 6). These results indicate that it is the cardiac sympathetic nerve rather than vagus mediates the tachycardia effect induced by microinjection of CRF into the PH.

### Responses of RVLM, RVMM, and DMNV neurons to microinjection of CRF into the PH

**Figure 7:**
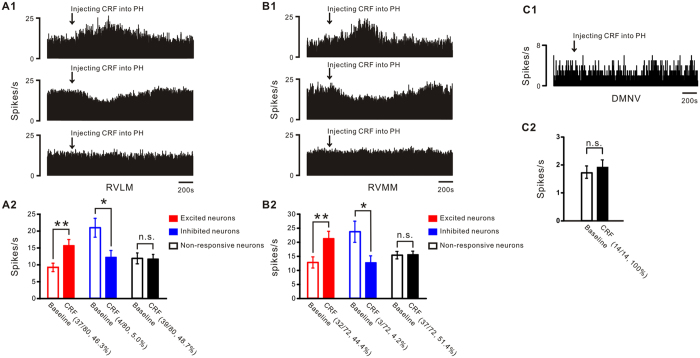
Responses of RVLM, RVMM and DMNV neurons to microinjection of CRF into the PH in rats. (**A1,A2**) The upper and middle peristimulus time histograms of A1 show two single RVLM neurons increased or decreased firing rate in response to microinjection of CRF into the PH, respectively. The lower histogram in A1 shows a non-responsive RVLM neuron to the microinjection. The group data with statistical results obtained from 80 recorded RVLM neurons is shown in A2. Note that 46.3% (37/80) RVLM neurons were excited. (**B1**,**B2**) RVMM neurons also showed increase, decrease, or no change in firing rate in response to microinjection of CRF into the PH, respectively. The layout of this panel is the same with (**A1,A2**). (**C1,C2**) Firing rate of DMNV neurons exhibited no change in response to microinjection of CRF into the PH. Statistical significance was determined using Student’s t test. n.s. no significance, *P < 0.05, **P < 0.01.

Considering that the RVLM and RVMM in the brainstem are two most important areas of presympathetic neurons, whereas the DMNV and nucleus ambiguus are two nuclei gathering vagal preganglionic neurons^28^, we conducted extracellular recordings in the RVLM, RVMM and DMNV (the nucleus ambiguus neurons were not recorded since they are intrinsically silent^29^) *in vivo* to clarify which structures were involved in the tachycardic effect elicited by microinjection of CRF into the PH. We recorded 80 RVLM, 72 RVMM and DMNV neurons in rats (*n* = 12). Among them, 37 (37/80, 46.3%) RVLM neurons and 32 (32/72, 44.4%) RVMM neurons increased their firing rates from 9.3 ± 1.2 spikes/s and 12.8 ± 1.9 spikes/s to 15.7 ± 1.8 spikes/s (*P* < 0.01) and 21.3 ± 2.5 spikes/s (*P* < 0.01), respectively, in response to microinjection of CRF into the PH (Fig. 7). In addition, 4 (4/80, 5.0%) RVLM neurons and 3 (3/72, 4.2%) RVMM neurons decreased their firing rates from 21.0 ± 2.8 spikes/s and 23.8 ± 3.8 spikes/s to 12.3 ± 2.0 spikes/s (*P* < 0.05) and 12.8 ± 2.4 spikes/s (*P* < 0.05), respectively, to microinjection of CRF into the PH (Fig. 7), and the rest 39 RVLM neurons and 37 RVMM neurons showed no response. On the other hand, all of the 14 recorded DMNV neurons had no response (1.9 ± 0.3 spikes/s *v.s.* 1.7 ± 0.2 spikes/s, *P* = 0.58) to microinjection of CRF into the PH (Fig. 7). The results suggest that it is the RVLM and RVMM but not DMNV mediate the tachycardia effect induced by microinjection of CRF into the PH.

## Discussion

CRF holds a key position in regulating and integrating various stress responses^1^,^2^ and the linkage between stress and cardiovascular disease risk has attracted a growing attention^3^,^4^,^30^. However, mechanism of central regulation of CRF on cardiovascular activities remains enigmatic. Here, we demonstrate a novel contribution of the PH, a pivotal structure responsible for generation of stress-induced cardiovascular responses, in the central effect of CRF on cardiovascular activities. CRFR1 and CRFR2 are expressed and co-localized in the same neurons in PH in rats. Through activation of both CRFR1 and CRFR2, CRF directly excites the PH neurons and actively elevates HR rather than AP. Furthermore, the tachycardia effect induced by microinjecting CRF into the PH is mediated by PH-RVLM/RVMM-cardiac sympathetic nerve pathway rather than PH-DMNV-vagus nerve pathway.

CRFR1 and/or CRFR2 are found to be expressed in many areas of cardiovascular centers including the hypothalamic paraventricular nucleus, arcuate nucleus, and nucleus tractus solitarii, nucleus ambiguus, and RVLM in brainstem^31^,^32^,^33^,^34^,^35^,^36^,^37^. Via coupling to various ionic mechanisms, CRFR1 and CRFR2 may mediate different effect of CRF related peptides on these neurons. For instance, CRFR1 and its coupled hyperpolarization-activated cation channel mediate the CRF-induced excitation^38^, whereas CRFR2 and its linked G protein-activated inwardly rectifying potassium channel mediate the urocortin 3-evoked inhibition on paraventricular nucleus neurons^39^. By these ways, both of CRF receptors may actively participate in central modulation of cardiovascular activities^31^,^32^,^33^,^34^,^35^,^36^,^37^. In the present study, we report, for the first time, that CRFR1 and CRFR2 are co-expressed in the PH and co-mediate the CRF-induced excitation on PH neurons (Figs 1, 2, 3). Since either excitation by electrical stimulation or disinhibition by microinjection GABA receptor antagonist in the PH significantly increased AP and HR^19^,^40^, our result that CRF excited the PH neurons suggests a possibility that PH may contribute to central regulation of CRF on cardiovascular activities. Intriguingly, microinjection of CRF into the PH significantly increased HR but not MAP and RSNA (Fig. 4), suggesting that by activating PH neurons, CRF may actively affect cardiac activity. Furthermore, selective CRFR1 antagonist antalarmin or selective CRFR2 antagonist antisauvagine-30 partly decreased tachycardiac response to microinjecting CRF into the PH, while broad-spectrum CRF receptor antagonist astressin totally blocked the response (Fig. 5), demonstrating that the tachycardiac response to CRF microinjected into the PH is mediated by activation of both CRFR1 and CRFR2. Nonetheless, microinjection of selective antagonist for CRFR1 or CRFR2 or broad-spectrum CRF receptor antagonist into the PH before CRF injection did not elicit any changes in basal HR (Fig. 5A). Thus, we speculate that under normal physiological conditions, CRF receptors in the PH may be not under a status of tonic control of endogenous CRF to modulate cardiovascular activity.

Microinjection of CRF into the PH only elevated HR but did not influence AP and RSNA suggests that the regulation of cardiac activity by CRF may be mediated by autonomic cardiovascular centers. On the basis of current knowledge regarding the pathway underlying the PH control of cardiac function, activation of cardiac sympathetic nerve or inhibition of cardiac vagal nerve may be responsible for tachycardiac effect followed by microinjection of CRF into the PH. In this study, we found that vagotomy did not affect the tachycardiac response by CRF, while blockade of CSNA with propranolol almost totally abolished the tachycardiac response (Fig. 6). These results reveal that the tachycardia response is not an effect of reduced vagus nerve activity elicited by microinjecting CRF into PH, but due to an increase in CSNA. This result accords with the previous reports that activation of the PH increased HR by increasing sympathetic nerve activity^41^,^42^. It is well known that the PH neurons project to various areas/structures in the brainstem, which are involved in regulation of cardiovascular activity, including the RVLM and RVMM^43^. The RVLM and RVMM are the key centers providing synaptic inputs to sympathetic preganglionic neurons and directly regulating sympathetic activity to influence cardiovascular function. Excitation of RVLM^44^ and RVMM^45^ increases HR and AP by strengthening sympathetic nerve activity which output through the sympathetic preganglionic neurons of intermediolateral cell column in spinal cord. Thus, the tachycardiac effect induced by microinjection of CRF in the PH may be mediated by the excitation of RVLM and RVMM neurons. In fact, in this study, we found that it is the RVLM and RVMM neurons rather than DNV neurons controlling vagus activity responsive to microinjecting CRF into the PH. Therefore, we speculate that the pathways from the PH to the RVLM and RVMM may contribute to the modulation of central CRF system on sympathetic outputs to the heart and the consequent HR regulation.

Tachycardia is an important component and feature of stress-induced cardiovascular responses and dysfunctions. We demonstrate that CRF, a critical neuropeptide responsible for initiating and integrating stress responses, induces a remarkable tachycardia by exciting PH neurons, which has been proposed to participate in the generation of stress-induced tachycardia^23^, through both CRFR1 and CRFR2. Pathways from the PH to the RVLM and RVMM, which subsequently regulate sympathetic outflow, may contribute to the tachycardia induced by activation of PH by CRF. The findings not only help to understand the effect and mechanism of central CRF on cardiovascular regulation, but also provide a new insight into the stress-related cardiovascular dysfunctions and disorders.

## Methods

### Animals

We used male Sprague-Dawley rats (375 ± 50 g), housed under controlled conditions with a lighting schedule of 12 h light and 12 h dark at a temperature of 22 ± 2 °C. Standard food and water were provided *ad libitum*. All experiments, approved by the Experimental Animal Care and Use Committee of Nanjing University, were treated in accordance with U.S. National Institutes of Health Guide for the Care and Use of Laboratory Animals (NIH Publication 85-23, revised 2011) and were reported in accordance with the ARRIVE guidelines^46^. All efforts were made to minimize the number of animals used and their suffering.

### Quantitative real-time RT-PCR

For this experiment, three independent groups of RNA pools each from 5 rats, deeply anaesthetized with urethane (1600 mg/kg) plus α-chloralose (130 mg/kg) given intraperitoneally, were used as biological replicates. The PH tissue punches were collected from coronal brain slices of rats according to the rat brain atlas of Paxinos and Watson^25^ and pooled (5 animals in each pool). RNA extraction was done using an RNA isolation kit (Invitrogen, USA) according to the manufacturer’s instructions. The reaction was carried out in an Applied Biosystem 7300 Real-time PCR System using the following parameters: 47 cycles at 95 °C for 5 s, 60 °C for 30 s, 95 °C for 15 s, 55 °C for 30 s and 95 °C for 15 s. The PCR program was completed by a melting temperature analysis. For quantification, the quantity of the target gene (*crfr1*and *crfr2*) was expressed relative to the amount of the reference gene (*gapdh*) to obtain a normalized target expression value.

The primer sequences were as follows: *crfr1*: forward, 5′-TTC TAC GGT GTC CGC TAC-3′ and reverse, 5′-CGA GAT GAG GTT CCA GTG -3′; *crfr2*: forward, 5′-CCT GCC CTA TCA TTG TCG-3′ and reverse, 5′-CCT TCA CTG CCT TCC TGT-3′; *gapdh*: forward, 5′-TTC AAC GGC ACA GTC AAG G-3′ and reverse, 5′-CTC AGC ACC AGC ATC ACC-3′.

### Immunofluorescence

The experimental procedures for immunostaining followed our previous reports^47^,^48^. Briefly, rats (*n* = 5) were deeply anesthetized and the rat brain was removed and fixed in the 4% paraformaldehyde in 0.1 M phosphate buffer for 12 h at 4 °C, and then cryoprotected with 30% sucrose for 48 h. Frozen coronal sections (25 μm thickness) containing the PH were obtained by using a freezing microtome (CM 1850, Leica, Germany). The slices were rinsed in phosphate-buffered saline containing 0.1% Triton X-100 (PBST) (Sigma, USA) and then incubated in 10% normal bovine serum (Millipore, USA) in PBST for 30 min. Sections were incubated overnight at 4 °C with primary antibodies to CRFR1 (a rabbit anti-CRFR1 polyclonal antibody, 1:200; Abcam, USA) and CRFR2 (a goat anti-CRFR2 polyclonal antibody, 1:200; Abcam). After a complete wash in PBS, sections were incubated in the Alexa 594-conjugated donkey anti-rabbit (1:2,000; Invitrogen) and Alexa 488-conjugated donkey anti-goat (1:2,000; Invitrogen) for 2 h at room temperature in the dark. The slides were washed and mounted in UltraCruz mounting medium (Santa Cruz Biotechnology, USA). All micrographs were taken with an inverted laser scanning confocal microscope (FV1000; Olympus, Japan).

### Patch clamp recordings on brain slices

Rats aged 14–21 days were deeply anaesthetized and coronal brainstem slices (300 μm in thickness) containing the PH were prepared with a vibroslicer (VT 1200 S, Leica), according to the rat brain atlas^25^. The slices were incubated in artificial cerebrospinal fluid (ACSF, composition in mM: 124 NaCl, 2.5 KCl, 1.25 NaH_2_PO_4_, 1.3 MgSO_4_, 26 NaHCO_3_, 2 CaCl_2_ and 10 D-glucose) equilibrated with 95% O_2_ and 5% CO_2_ at 35 ± 0.5 °C for at least 1 hour and then maintained at room temperature. During recording sessions, the slices were transferred to a submerged chamber and continuously superfused with 95% O_2_ and 5% CO_2_ oxygenated ACSF at a rate of 2 ml/min maintained at room temperature.

Whole-cell recordings were performed as we previously described^47^,^48^. Briefly, PH neurons were visualized with an Olympus BX51WI microscope and then recorded with borosilicate glass pipettes (3–5 MΩ) filled with an internal solution (composition in mM: 140 K-methylsulfate, 7 KCl, 2 MgCl_2_, 10 HEPES, 0.1 EGTA, 4 Na_2_-ATP, 0.4 GTP-Tris, adjusted to pH 7.25 with 1 M KOH). Patch clamp recordings were acquired with an Axopatch-700B amplifier (Axon Instruments, USA) and the signals were fed into a computer through a Digidata-1550 interface (Axon Instruments) for data capture and analysis (pClamp 10.0, Axon Instruments).

We bathed the slices with CRF (0.03–3 μM, Millipore) to stimulate the recorded PH neurons. TTX (0.3 μM, Alomone Labs, Israel) combining with DNQX (30 μM, Tocris, UK), AP5 (50 μM, Tocris, UK) and SR 95531 (50 μM, Tocris, UK) was used to determine whether the effect of CRF is postsynaptic. Selective antagonists for CRFR1 and CRFR2, antalarmin (300 nM, Tocris, UK) and antisavagine-30 (100 nM, Tocris), respectively, as well as selective agonists for the two receptors, stressin I (1 μM, Tocris) and urocortin II (1 μM, Phoenix pharmaceuticals, USA), respectively, were applied to assess the underlying receptor mechanism.

### Cardiovascular activities recording *in vivo*

Rats were anaesthetized with urethane (800 mg/kg) plus α-chloralose (65 mg/kg) given intraperitoneally and maintained at surgical depth throughout the experimental session. Supplemental doses of urethane plus α-chloralose were given intravenously when necessary if nociceptive stimulation of hind paw (tested every half an hour) caused a change in MAP of more than 10 mmHg. Rectal temperature was maintained at 37 ± 0.5 °C. The trachea was intubated for artificial respiration. The right carotid artery was cannulated with polyethylene tubing (i.d. 0.5 mm, o.d. 0.9 mm) filled with normal saline containing heparin (30 U/ml, Sigma, USA) for the measurement of AP. The polyethylene tubing was connected to a physiological pressure amplifier (Bridge Amp, ADInstruments, Australia) through a transducer (SP844, ADInstruments). MAP and HR were derived from the AP tracing. All signals were acquired online using LabChart 7 software (ADInstruments). The left renal sympathetic nerve was isolated and cut distally to permit recording of efferent RSNA. Nerve recordings were made with bipolar silver wire electrodes. The renal nerve branch and the electrode were immersed in warm liquid paraffin. The signals from the recording electrodes were amplified (×1000) and filtered (bandwidth 100–2,000 Hz). These signals were recorded on a computer using a PowerLab system (ADInstruments). LabChart 7 software was used to rectify and integrate the renal sympathetic nerve signals.

Bilateral cervical vagus nerves were isolated and tied with silk threads. The vagus nerves were cut by carefully pulling on the silk loop to detect the CRF-induced changes in HR after bilateral vagotomy followed a stabilization period of 15–20 min. To assess role of cardiac sympathetic nerve in the CRF-induced changes in HR, the jugular vein was cannulated for administrating propranolol (1 mg/kg, Sigma) to block the cardiac sympathetic nerve activity (CSNA).

### Microinjection in the PH

The rat was mounted on a stereotaxic frame (1404, David Kopf Instruments, USA). The craniotomy was made above the PH. According to the rat brain atlas^25^, the stainless-steel injection cannulae (length 10 mm, o.d. 0.5 mm, i.d. 0.3 mm) were inserted into the PH (A −3.60 to −3.96, L 0.5, H 7.5) for microinjection of CRF (20–200 μM), astressin (300 μM), antalarmin (500 μM), antisauvagine-30 (500 μM), and saline (0.9% NaCl) using Hamilton syringes (0.5 μl, lasting 2 min). The effective extent of the drug diffusion in the present study was restricted in the PH according to the estimate by using extracellular electrophysiological recording neurons 0.5 mm away from the injection site (the detailed descriptions of methodology can be obtained from our previous reports^47^,^48^). The effects of CRF on HR, MAP and RSNA before and after microinjections were detected.

### Extracellular recording in brainstem *in vivo*

Single neuronal discharges were recorded from the RVLM (A −12.00 to −12.48, L 1.8 to 2.4, H 8.0 to 8.5), RVMM (A −10.00 to −12.20, L 0.0 to 0.6, H 8.5 to 9.5), and DMNV (A −13.08 to −13.20, L 0.5 to 1.0, H 5.2 to 5.5), according to the rat brain atlas^25^, by using a glass electrode filled with 1% solution of pontamine sky blue in 0.5 M sodium acetate (DC resistance 5–10 MΩ). The neuronal discharges were conventionally amplified, displayed on an oscilloscope, and simultaneously fed into a window discriminator. The standard rectangular pulses (1.0 ms, 5 V) triggered from the spikes were sent through an A/D interface (Power 1401, CED, UK) into a laboratory computer.

The peristimulus time histogram (sampling interval 1 sec, sampling length 1,800 sec, single trial) of neuronal discharges were generated by the software Spike 2 (CED) to assess the effects of microinjection of CRF into PH on neuronal discharges of RVLM, RVMM, and DMNV, respectively. According to the previous established criteria^49^,^50^, any significant change in neuronal discharge rate, reproducibly evoked by the microinjection, with *P* < 0.05 was considered as an excitatory or inhibitory response. After experiments, microinjection sites in the PH and recording sites in the RVLM, RVMM, and DMNV were histologically identified as we previously reported^47^,^48^,^49^,^50^. Data from rats in which the injection and the recording sites were out of the target nucleus were excluded from further analysis.

### Statistical analysis

All data are presented as mean ± SEM. Preliminary tests for normality of each variable were executed. The Student’s t test was employed for statistical analysis. *P* < 0.05 was considered significant.

## Additional Information

**How to cite this article**: Gao, H.-R. *et al.* Corticotropin releasing factor excites neurons of posterior hypothalamic nucleus to produce tachycardia in rats. *Sci. Rep.* **6**, 20206; doi: 10.1038/srep20206 (2016).

## References

[b1] Vale W., Spiess J., Rivier C. & Rivier J. Characterization of a 41-residue ovine hypothalamic peptide that stimulates secretion of corticotropin and beta-endorphin. Science 213, 1394–1397 (1981). 6267699 10.1126/science.6267699

[b2] Bale T. L. & Vale W. W. CRF and CRF receptors: role in stress responsivity and other behaviors. Annu Rev Pharmacol Toxicol 44, 525–557, doi: 10.1146/annurev.pharmtox.44.101802.121410 (2004). 14744257

[b3] Schubert C. *et al.* Effects of stress on heart rate complexity–a comparison between short-term and chronic stress. Biol Psychol 80, 325–332, doi: 10.1016/j.biopsycho.2008.11.005 (2009). 19100813 PMC2653595

[b4] Dimsdale J. E. Psychological stress and cardiovascular disease. J Am Coll Cardiol 51, 1237–1246, doi: 10.1016/j.jacc.2007.12.024 (2008). 18371552 PMC2633295

[b5] Thayer J. F., Yamamoto S. S. & Brosschot J. F. The relationship of autonomic imbalance, heart rate variability and cardiovascular disease risk factors. Int J Cardiol 141, 122–131, doi: 10.1016/j.ijcard.2009.09.543 (2010). 19910061

[b6] Jin R. *et al.* Intracerebroventricular injection of stresscopin-related peptide enhances cardiovascular function in conscious rats. Regul Pept 186, 7–11, doi: 10.1016/j.regpep.2013.07.001 (2013). 23850799

[b7] Fisher L. A. *et al.* Corticotropin-releasing factor (CRF): central effects on mean arterial pressure and heart rate in rats. Endocrinology 110, 2222–2224, doi: 10.1210/endo-110-6-2222 (1982). 6978810

[b8] Briscoe R. J., Cabrera C. L., Baird T. J., Rice K. C. & Woods J. H. Antalarmin blockade of corticotropin releasing hormone-induced hypertension in rats. Brain Res 881, 204–207 (2000). 11036160 10.1016/s0006-8993(00)02742-6

[b9] Vaughan J. *et al.* Urocortin, a mammalian neuropeptide related to fish urotensin I and to corticotropin-releasing factor. Nature 378, 287–292, doi: 10.1038/378287a0 (1995). 7477349

[b10] Calderon-Sanchez E. *et al.* Urocortin induces positive inotropic effect in rat heart. Cardiovasc Res 83, 717–725, doi: 10.1093/cvr/cvp161 (2009). 19460778

[b11] Smani T. *et al.* Mechanisms underlying the activation of L-type calcium channels by urocortin in rat ventricular myocytes. Cardiovasc Res 87, 459–466, doi: 10.1093/cvr/cvq063 (2010). 20189952

[b12] Chen R., Lewis K. A., Perrin M. H. & Vale W. W. Expression cloning of a human corticotropin-releasing-factor receptor. Proc Natl Acad Sci USA 90, 8967–8971 (1993). 7692441 10.1073/pnas.90.19.8967 PMC47482

[b13] Perrin M. H., Donaldson C. J., Chen R., Lewis K. A. & Vale W. W. Cloning and functional expression of a rat brain corticotropin releasing factor (CRF) receptor. Endocrinology 133, 3058–3061, doi: 10.1210/endo.133.6.8243338 (1993). 8243338

[b14] Lovenberg T. W. *et al.* Cloning and characterization of a functionally distinct corticotropin-releasing factor receptor subtype from rat brain. Proc Natl Acad Sci USA 92, 836–840 (1995). 7846062 10.1073/pnas.92.3.836 PMC42715

[b15] Dautzenberg F. M. & Hauger R. L. The CRF peptide family and their receptors: yet more partners discovered. Trends Pharmacol Sci 23, 71–77 (2002). 11830263 10.1016/s0165-6147(02)01946-6

[b16] Martinez-Laorden E. *et al.* Corticotropin-releasing factor (CRF) receptor-1 is involved in cardiac noradrenergic activity observed during naloxone-precipitated morphine withdrawal. Br J Pharmacol 171, 688–700, doi: 10.1111/bph.12511 (2014). 24490859 PMC3969081

[b17] Bale T. L. *et al.* Mice deficient for corticotropin-releasing hormone receptor-2 display anxiety-like behaviour and are hypersensitive to stress. Nat Genet 24, 410–414, doi: 10.1038/74263 (2000). 10742108

[b18] Coste S. C. *et al.* Abnormal adaptations to stress and impaired cardiovascular function in mice lacking corticotropin-releasing hormone receptor-2. Nat Genet 24, 403–409, doi: 10.1038/74255 (2000). 10742107

[b19] Barron K. W. & Heesch C. M. Cardiovascular effects of posterior hypothalamic stimulation in baroreflex-denervated rats. Am J Physiol 259, H720–727 (1990). 2396685 10.1152/ajpheart.1990.259.3.H720

[b20] Martin J. R. Neuropeptide Y potentiates the pressor response evoked by carbachol administration into the posterior hypothalamic nucleus of conscious rat. Brain Res 949, 79–87 (2002). 12213302 10.1016/s0006-8993(02)02967-0

[b21] Iyoda I. *et al.* Cardiovascular and sympathetic responses to ouabain injected into the hypothalamus in rats. Cardiovasc Res 20, 294–298 (1986). 3719610 10.1093/cvr/20.4.294

[b22] Cortelli P. *et al.* Effect of deep brain stimulation of the posterior hypothalamic area on the cardiovascular system in chronic cluster headache patients. Eur J Neurol 14, 1008–1015, doi: 10.1111/j.1468-1331.2007.01850.x (2007). 17718693

[b23] Lisa M., Marmo E., Wible J. H. Jr. & DiMicco J. A. Injection of muscimol into posterior hypothalamus blocks stress-induced tachycardia. Am J Physiol 257, R246–251 (1989). 2750964 10.1152/ajpregu.1989.257.1.R246

[b24] Lisa M., Filippelli A., Marmo E., Wible J. H. Jr. & DiMicco J. A. Microinjection of muscimol into posterior hypothalamus blocks cardiovascular response to experimental stress in rats. Pharmacol Res 21 Suppl 1, 9–10 (1989). 2633198 10.1016/s1043-6618(89)80027-1

[b25] Paxinos G. & Watson C. The rat brain in stereotaxic coordinates. 6th edn, (Academic Press/Elsevier, 2007).

[b26] Myers B. *et al.* GABAergic Signaling within a Limbic-Hypothalamic Circuit Integrates Social and Anxiety-Like Behavior with Stress Reactivity. Neuropsychopharmacology, doi: 10.1038/npp.2015.311 (2015). PMC4832014 26442601

[b27] Ikemoto S., Witkin B. M., Zangen A. & Wise R. A. Rewarding effects of AMPA administration into the supramammillary or posterior hypothalamic nuclei but not the ventral tegmental area. J Neurosci 24, 5758–5765, doi: 10.1523/JNEUROSCI.5367-04.2004 (2004). 15215298 PMC6729211

[b28] Llewellyn-Smith I. J. & Verberne A. J. M. Central regulation of autonomic functions. 2nd edn, (Oxford University Press, 2011).

[b29] Mendelowitz D. Firing properties of identified parasympathetic cardiac neurons in nucleus ambiguus. Am J Physiol 271, H2609–2614 (1996). 8997322 10.1152/ajpheart.1996.271.6.H2609

[b30] Steptoe A. & Kivimaki M. Stress and cardiovascular disease. Nat Rev Cardiol 9, 360–370, doi: 10.1038/nrcardio.2012.45 (2012). 22473079

[b31] Milner T. A., Reis D. J., Pickel V. M., Aicher S. A. & Giuliano R. Ultrastructural localization and afferent sources of corticotropin-releasing factor in the rat rostral ventrolateral medulla: implications for central cardiovascular regulation. J Comp Neurol 333, 151–167, doi: 10.1002/cne.903330203 (1993). 7688383

[b32] Chitravanshi V. C. & Sapru H. N. Microinjections of urocortin1 into the nucleus ambiguus of the rat elicit bradycardia. Am J Physiol Heart Circ Physiol 300, H223–229, doi: 10.1152/ajpheart.00391.2010 (2011). 20952663 PMC3023261

[b33] Chitravanshi V. C., Kawabe K. & Sapru H. N. Bradycardic effects of microinjections of urocortin 3 into the nucleus ambiguus of the rat. Am J Physiol Regul Integr Comp Physiol 303, R1023–1030, doi: 10.1152/ajpregu.00224.2012 (2012). 23019211 PMC3517671

[b34] Nakamura T., Kawabe K. & Sapru H. N. Cardiovascular responses to microinjections of urocortin 3 into the nucleus tractus solitarius of the rat. Am J Physiol Heart Circ Physiol 296, H325–332, doi: 10.1152/ajpheart.01044.2008 (2009). 19060121 PMC2643898

[b35] Chitravanshi V. C., Kawabe K. & Sapru H. N. Mechanisms of cardiovascular actions of urocortins in the hypothalamic arcuate nucleus of the rat. Am J Physiol Heart Circ Physiol 305, H182–191, doi: 10.1152/ajpheart.00138.2013 (2013). 23686711 PMC3726959

[b36] Li X. *et al.* Excitatory responses of cardiovascular activities to urocortin3 administration into the PVN of the rat. Auton Neurosci 154, 108–111, doi: 10.1016/j.autneu.2009.12.004 (2010). 20060787

[b37] Yamazaki T. *et al.* Microinjection of urocortin into the rat nucleus tractus solitarii decreases arterial blood pressure. Auton Neurosci 142, 51–54, doi: 10.1016/j.autneu.2008.07.013 (2008). 18804421

[b38] Qiu D. L. *et al.* Corticotrophin-releasing factor augments the I(H) in rat hypothalamic paraventricular nucleus parvocellular neurons *in vitro*. J Neurophysiol 94, 226–234, doi: 10.1152/jn.01325.2004 (2005). 15800070

[b39] Chu C. P. *et al.* Effects of stresscopin on rat hypothalamic paraventricular nucleus neurons *in vitro*. PLoS One 8, e53863, doi: 10.1371/journal.pone.0053863 (2013). 23349753 PMC3548845

[b40] Shonis C. A., Peano C. A., Dillon G. H. & Waldrop T. G. Cardiovascular responses to blockade of GABA synthesis in the hypothalamus of the spontaneously hypertensive rat. Brain Res Bull 31, 493–499 (1993). 8495374 10.1016/0361-9230(93)90115-r

[b41] DiMicco J. A. & Abshire V. M. Evidence for GABAergic inhibition of a hypothalamic sympathoexcitatory mechanism in anesthetized rats. Brain Res 402, 1–10 (1987). 3828776 10.1016/0006-8993(87)91041-9

[b42] Wible J. H. Jr., Luft F. C. & DiMicco J. A. Hypothalamic GABA suppresses sympathetic outflow to the cardiovascular system. Am J Physiol 254, R680–687 (1988). 3354717 10.1152/ajpregu.1988.254.4.R680

[b43] Vertes R. P. & Crane A. M. Descending projections of the posterior nucleus of the hypothalamus: Phaseolus vulgaris leucoagglutinin analysis in the rat. J Comp Neurol 374, 607–631, doi: 10.1002/(SICI)1096-9861(19961028)374:4&lt;607::AID-CNE9&gt;3.0.CO;2-5 (1996). 8910738

[b44] Ross C. A., Ruggiero D. A., Joh T. H., Park D. H. & Reis D. J. Rostral ventrolateral medulla: selective projections to the thoracic autonomic cell column from the region containing C1 adrenaline neurons. J Comp Neurol 228, 168–185, doi: 10.1002/cne.902280204 (1984). 6480910

[b45] Babic T. & Ciriello J. Medullary and spinal cord projections from cardiovascular responsive sites in the rostral ventromedial medulla. J Comp Neurol 469, 391–412, doi: 10.1002/cne.11024 (2004). 14730590

[b46] Kilkenny C., Browne W. J., Cuthill I. C., Emerson M. & Altman D. G. Improving bioscience research reporting: the ARRIVE guidelines for reporting animal research. PLoS Biol 8, e1000412, doi: 10.1371/journal.pbio.1000412 (2010). 20613859 PMC2893951

[b47] Zhang J. *et al.* A role for orexin in central vestibular motor control. Neuron 69, 793–804, doi: 10.1016/j.neuron.2011.01.026 (2011). 21338887

[b48] Zhang J. *et al.* Selective Modulation of Histaminergic Inputs on Projection Neurons of Cerebellum Rapidly Promotes Motor Coordination via HCN Channels. Mol Neurobiol, doi: 10.1007/s12035-015-9096-3 (2015). 25633097

[b49] Wen Y. Q., Zhu J. N., Zhang Y. P. & Wang J. J. Cerebellar interpositus nuclear inputs impinge on paraventricular neurons of the hypothalamus in rats. Neurosci Lett 370, 25–29, doi: 10.1016/j.neulet.2004.07.072 (2004). 15489011

[b50] Li B., Guo C. L., Tang J., Zhu J. N. & Wang J. J. Cerebellar fastigial nuclear inputs and peripheral feeding signals converge on neurons in the dorsomedial hypothalamic nucleus. Neurosignals 17, 132–143, doi: 10.1159/000197913 (2009). 19182493

